# Is Cancer Reversible? Rethinking Carcinogenesis Models—A New Epistemological Tool

**DOI:** 10.3390/biom13050733

**Published:** 2023-04-24

**Authors:** Andrea Pensotti, Marta Bertolaso, Mariano Bizzarri

**Affiliations:** 1 Research Unit of Philosophy of Science and Human Development, University Campus Bio-Medico of Rome, 00128 Rome, Italy; 2 Systems Biology Group Lab, Department of Experimental Medicine, Sapienza University, 00185 Rome, Italy

**Keywords:** tumor reversion, microenvironment, embryo development, systems biology, morphogenesis, biological network

## Abstract

A growing number of studies shows that it is possible to induce a phenotypic transformation of cancer cells from malignant to benign. This process is currently known as “tumor reversion”. However, the concept of reversibility hardly fits the current cancer models, according to which gene mutations are considered the primary cause of cancer. Indeed, if gene mutations are causative carcinogenic factors, and if gene mutations are irreversible, how long should cancer be considered as an irreversible process? In fact, there is some evidence that intrinsic plasticity of cancerous cells may be therapeutically exploited to promote a phenotypic reprogramming, both in vitro and in vivo. Not only are studies on tumor reversion highlighting a new, exciting research approach, but they are also pushing science to look for new epistemological tools capable of better modeling cancer.

## 1. Introduction

The first clinical evidence of spontaneous cancer regression came from teratocarcinoma. In 1907, the Swiss pathologist, Max Askanazy, observed the spontaneous reversion of an ovarian teratocarcinoma [1]. A more thorough study of these processes was possible in 1954 after Stevens and Little’s work on the 129/SvJ mouse had led to a model of teratocarcinoma [2].

In 1959, Pierce made his first observations on the spontaneous differentiation of embryonal cancer cells deriving from testicular teratocarcinoma in the 129/SvJ mouse [3]. Pierce highlighted the pivotal role of the cell microenvironment and introduced the hypothesis of “the development of methods that would direct the differentiation of embryonal carcinoma cells to benign forms as a logical means of controlling this type of cancer” [4]. These results were instrumental in introducing the concept of “cancer reversion”, indicating the recovery of a normal phenotype by cancerous cells when exposed to a specific microenvironment.

In 1974, Brinster confirmed Pierce’s hypothesis. He injected testicular teratocarcinoma cells from the 129/SvJ black agouti into murine blastocysts, and then implanted these blastocysts into albino female mice. This resulted in a healthy black-white hybrid offspring, suggesting that “the embryo environment can bring under control the autonomous proliferation of the teratocarcinoma cells” [5]. Similar results were obtained by Mintz and Illmensee. They had injected embryonal cancer cells into 280 different blastocysts that were further implanted into as many adoptive mothers. Both analyses on the fetus and the offspring showed no signs of cancer cells. Even more interesting were the results of the analyses on the composition of hair, the type of circulating red and white blood cells, the protein composition of urine, and the characteristics of the kidneys, liver, and thymus. From all of these analyses, it emerged that the teratocarcinoma cells deriving from the 129/SvJ black agouti mice participated in the normal formation of the organs by integrating “in mosaic” with the cells of the brown C57-b/b mouse strain. Following these results, Mintz and Illmensee concluded:

*“The capacity of embryonal carcinoma cells to form normally functioning adult tissues demonstrates that conversion to neoplasia did not involve structural changes in the genome, but rather a change in gene expression”*.

[6]

Subsequent experiments demonstrated the role of the embryonic microenvironment in controlling the proliferation of different cancer types such as Rous sarcoma [7], leukemia, neuroblastoma [8], melanoma [9,10], colon, and breast [11,12], to mention a few.

Despite this experimental evidence, the “reversion” approach in cancer research remains insufficiently explored. One reason may be in the epistemological tools needed to model reversion processes: experimental results on tumor reversion need a systemic approach in cancer modeling. This approach forces us to reconsider entire cancer models developed under a mainly reductionist approach. The aim of this article is, therefore, to investigate how the epistemological tools that we need to model tumor reversion processes may reshape our perspective on cancer and offer new heuristic models for cancer research. Rethinking the carcinogenic model is mandatory to plan a very different strategy in cancer treatment. During the last 40 years, the survival rate of patients suffering from different kinds of cancer has increased up to 20%. This is an appreciable result, although it mostly depended on early diagnosis and the development of improved surgical and radiation-based therapies, while medical treatments provided only marginal benefits in solid cancers [13]. A similar perspective is emerging regarding target-based drugs, which show a limited curative potential [14].

## 2. Tumor Reversion—An Experimental Model Overview

Different experimental models [15] have proven that, in certain circumstances, cancer cells can revert their phenotype from malignant to benign. A systematic literature review shows the emergence of the main experimental models. Schematically, these can be classified as follows: (a) observation of spontaneous in vivo cancer regressions; (b) in vivo model of cancer cells grafted into normal tissues; (c) in vivo model of cancer cells grafted into blastocyst, and (d) in vitro model of cancer reversion following the exposure of tumor cells to embryonal microenvironmental factors or embryonic stem cell factors. The different variables taken into consideration in these models are: (I) type of tumor; (II) specific phase of the embryonic development of the organism in which the tumor is implanted, and (III) anatomical site of the tumor graft. Table 1 recapitulates the most relevant studies published in the field.

Besides the complexity of the experimental and interpretative framework, some conclusions from the most recurrent elements can be drawn, namely:

(a) Cancer cells display relevant plasticity, and their fate is not “irreversibly” determined.
(b) It is possible to inhibit the phenotypic expression of the malignant characteristics of cancer cells mostly through epigenetic processes, although other mechanisms are likely to participate.
(c) Depending on the tumor type and stage, some context-dependent conditions/constraints (such as those pertaining to the microenvironment of specific embryogenesis stages) can induce a phenotypic reversion of malignant cancer cells.
(d) Gene mutations do not play a “causative” role as the somatic mutation theory (SMT) posits, albeit they can be associated throughout the process of cancer development.

Despite such experimental evidence, the concept of cancer reversibility has not been systematically explored. It is important to specify that reversibility does not mean the return of cancer cells to the original state. Rather, it indicates a phenotypic transformation during which cells lose their main malignant traits (migrating and invasive capabilities) and acquire a benign-like architecture. From a clinical point of view, this response can be associated with either regression or tumor dormancy. Eventually, fibrous transformation can occur.

These results have been mostly underestimated, chiefly because current cancer models essentially rely on the somatic mutation theory (SMT). Indeed, the SMT focuses on gene mutations as the primary causes of cancer and can hardly provide a convincing explanation of the reversion process.

Rather, the fact that cancer cells can undergo a phenotypic normalization without entailing their “mutational” status contradicts the very basic premises on which the SMT is grounded.

This draws attention to the concept of phenotypic expression, that is, the process mediated by a complex network of signals and biological mechanisms that govern DNA expression. For a long time, specific genes were associated with certain functions and characteristics expressed at a phenotypic level: “a gene–a function” had become a sort of axiom. Based on this approach, the SMT has implicitly excluded the concept of tumor reversion: if the malignant cell characteristics are the result of genetic mutations, then it is impossible to eliminate them without first correcting these coding errors [41].

However, the limits of the gene-centric view of cancer in accounting for certain experimental observations have already emerged.

Notable is this quote from an editorial published in Nature Magazine, which clearly states that “it urge(s) us to revisit the role of gene mutations in cancer (...) if not gene mutations, what else could cause cancer?” [42].

Such a question is also highlighted by Weinberg, who lucidly explained how *“the identities of mutant cancer genes varied considerably from one type of tumor to another (…) Each tumor seemed to represent a unique experiment of nature (…) We cannot really assimilate and interpret most of the data we accumulate. How is all this going to end? I wouldn’t pretend to know. It’s a job (…) for the next generation”* [43].

Weinberg also pointed out that “*the most potent carcinogens are actually not mutagens*” [43].

## 3. Critical Aspects of Gene-Centric Models

A number of published studies question the centrality of gene mutations in cancer-causing processes [44,45,46]. Moreover, it is becoming increasingly clear that not all oncogenes are mutagens [47,48], and not all tumors are associated with specific mutations. Unexpectedly, cancer can develop even in the absence of specific mutated genes [49,50,51]. For example, it has been observed that some oncogenes such as H-ras, N-ras, and K-ras are not clonal in the prostate [52], the colon [53] and in melanomas [54]. Mutations such as Her and EGFR, observed in tumors of the bladder [55] and the breast [56], as well as in gliomas [57], are also non-clonal. Surprisingly, certain mutations deemed to exert a causative role in malignant tumors are also detected in normal cells [58], while certain mutations affecting oncogenes and tumor suppressor genes often occur only in an advanced stage of cancer progression. In some cases, as in the case of EGFR changes, these mutations occur only in a limited number of tumor cells [59]. The same was observed for the oncogenes c-fos and c-erb B-3, which, paradoxically, turned out to be more frequent in healthy tissue cells than in colon cancer cells [60]. Other studies show that only 30–40% of cancer cells present genetic mutations [61], while mutations in genes highly correlated with tumors have been found in healthy cells [62]. Finally, some tumors do not present mutations at all [63,64].

These observations allow the scientific community to hypothesize that alterations in the “gatekeeper” and “caretaker” genes are not sufficient to initiate a tumor [65], and that perhaps the very hypothesis that cancer is the result of genetic mutations may be wrong [66,67].

The SMT derives from a reductionist approach. It moves from the assumption that in order to understand the biological systems, one needs to break them down into discrete entities, isolate each entity, and analyze them as if they were many small cogs of a complex machine. This assumption, also known as biological atomism, considers it possible to identify the elementary units, which, in principle, can explain any biological process [68].

Coherently with this model, cancer is interpreted as a complex phenomenon caused by a progressive accumulation of specific genetic mutations. Therefore, any understanding of oncogenic processes should be sought at the genetic level. Accordingly, current cancer research aims to identify the genetic footprint of each tumor, interpreted as the specific cause of cancer [69,70], by isolating cells and their DNA from the biological context. This approach, however, has to deal with the paradox that many of the mutations associated with cancer have also been found in healthy cells and that cells reverted from cancer status do not show any correction of mutated genes [71].

Cancer heterogeneity represents another critical issue: a tumor mass is composed of a heterogeneous population of cancer cells that, contrary to what was originally hypothesized by the monoclonal cancer origin model, show a different gene expression pattern as well as several relevant differences in phenotypic and behavioral traits [72,73,74]. Consequently, within an apparent homogenous population, clusters of cancer cells often respond differently to cancer treatments. In the most extreme case, genetic and phenotypic heterogeneity ultimately cooperate in promoting the selection of chemotherapy-resistant cancer cell lines [41,75,76].

These data suggest that it may be practically impossible to define a genetic fingerprint for each type of tumor, as the cancer genome is heterogenous and always changing across the disease progression. Consequently, the search for specific drug targets is a futile attempt. These targets, even when they are identified in a large fraction of the cancer cell population, could be numerous and constantly evolving, making it difficult to reach a definitive target-based solution for the treatment of cancer [77,78,79]. This might be the reason why the target-based therapy, i.e., the possibility of finding drugs capable of interfering exclusively with tumor cells carrying specific mutations [80], is currently largely questioned, due to disappointing results and the enormous imbalance between costs and results [81].

Generally, most of the recent anticancer drugs have not been able to make substantial contributions to the treatment of cancer patients. The best achievements performed along this path are actually limited to a six-month extension of the life expectancy of patients, with an average of around four months. In addition, many of these therapies fail to avoid relapses, which in most cases recur with greater malignancy than the primary tumor [41,82].

The aforementioned limits have been highlighted since the 1960s, when Barry Pierce, a pioneer in tumor reversion studies, expressed his concern about the hypothesis that cancer originated essentially from genetic mutations:

*“Most oncologists believe that insertion of viral information into the genome, or mutation (a structural change in the genome), is the underlying mechanism of carcinogenesis. On the basis of our experiences with spontaneously occurring embryonal carcinoma, and because all of the phenotypic traits of malignant cells appear to be encoded in the genome of normal cells, I favor the idea that the production of a neoplasm is probably similar to the production of any normal tissue (...) The mechanism of tissue genesis involves cell division, differentiation, and organization. In other words, I believe that carcinogenesis is an epigenetic event, similar to postembryonic differentiation”*.

[83]

Already then, in light of the experimental results obtained, he was able to trace a theoretical path that still needs to be deepened today:

*“If mutation proves to be the causative event, then our discovery that malignant cells can differentiate to benign cells implies that the process of differentiation is capable of regulating the mutation that causes cancer. If expression of an oncogene is the cause of cancer, then what we have shown is that the process of differentiation represses the oncogene”*.

[84]

When the role of genes and mutations within tumor processes is resized, then the theoretical-experimental framework of SMT is also reframed. Hence, there is a need to identify a new theoretical approach that can model the complexity inherent in the dynamic relationship between cells and (micro)environment that results in phenotypic expression processes. As Denis Noble explains, *“one cannot understand the physiology or the pathology of cardiac rhythm by only referring to the gene expression and to the features of a single cardiomyocyte”* [85].

Definitely, a theory is not neutral with respect to experimental data but plays an important role in determining what can be observed and, consequently, the setting of experimental models [86]. In other words, the interpretation of the same data can differ according to the theoretical frame we are embracing. Moreover, even the selection of relevant parameters and observables strictly depends on the theory, which is a prerequisite for any experimental endeavor. Even the most basic and simple empiricism relies on a set of a priori, i.e., fundamental theoretical premises within which experimental data should be nestled and interpreted [87].

Herein, what we would propose is, therefore, an interpretation of tumor reversion under a systemic view of cancer, i.e., the organicist approach.

## 4. The Role of the Microenvironment

The initial concept of the microenvironment was developed in the seed and soil theory by Paget at the end of the 19th century [88]. According to this theory, it was possible to explain the mechanisms of metastases as the product of favorable interactions between metastatic tumor cells, the “seed”, and their microenvironment, the “soil”. Nowadays, the cellular microenvironment means the environment surrounding the cell—a complex system composed of the extracellular matrix, capillaries, stroma cells (namely fibroblasts and immuno-competent cells), active substances (including cytokines and hormones) and many other diffusible molecular factors [89]. This whole set of elements, topographically positioned according to a balanced architecture, affects the cell by means of physical and biochemical pathways. In turn, cells can significantly perturb their microenvironment by releasing substances (collagen, fibronectin, metalloproteinases) that modify some critical features.

The pivotal role of the microenvironment in addressing cell fate is emerging also from studies performed in regenerative medicine and tissue bioengineering. These attempts seek to reproduce the processes of organogenesis by replicating what occurs in nature during embryonic development [90].

The cells, together with the tumor microenvironment, represent an integrated, dynamic system whose state is determined by the interactions of all of its components, modulated according to non-equilibrium thermodynamics. Genes are expressed through processes finely tuned by the gene regulatory network [91]. Examples of these mechanisms involve the micro-RNA-dependent post-transcriptional regulation and epigenetic control of gene expression [92].

However, the activity of gene and molecular regulation is not based only on signals that act at the local molecular level. It is also strongly modulated by signals and constraints that depend on the higher levels of biological organization [93]. The cells and the microenvironment are, therefore, essentially two entities that interact with each other. A higher level of observation shows that they constitute a single entity, i.e., an integrated system—the tissue that can, in turn, exert its influence onto lower levels.

Within this framework, the focus shifts from genes to the system, from single entities—cells and DNA—to the complex relationships between the components of the system. The most suitable tools for studying these interactions must, therefore, be sought in network and complex systems science.

### 4.1. Essentiality of the Microenvironment in Biological Models

In vitro experimental settings oversimplify the context within which genes and cells express their functions. In order to understand the logics that guide the dynamics of a system, we should pay attention not only to the single elements that compose it but also the context, that is, the microenvironment.

In this sense, a gene could be described not only by its specific nucleotide sequence but also its specific network of interactions, that is, its “connectivity” [94].

Undoubtedly, only an in vivo model can reproduce the entire relational context within which each single gene operates. The verification of the linear correspondence between gene and phenotypic characteristics is not always possible. On the contrary, unexpected effects due to the intricate network of gene expression pathways are often observed [95]. For example, the same pathological phenotype can be originated from different combinations of gene mutations [96]. 

The gap between the results of in vitro genetic studies and in vivo expression is still wide. It is clear that current therapeutic cancer strategies struggle to manage this complexity: different genes, in different contexts, can originate the same proteins, while the same genes can express themselves in different ways [78,97,98].

This is not meant to deny the relevance of genes and their specific sequences. However, there is a need to reinterpret their functioning in the context. The latter, in fact, is able to amplify, modulate, or inhibit the activity of each gene. Oncogenes and tumor suppressor genes inevitably also fall into this picture [99].

### 4.2. The Microenvironment as a Target

Some interesting experimental works have highlighted the role of the microenvironment in tumor processes and, more generally, in cell phenotypic commitment.

For instance, the carcinogen N-nitroso-methyl urea can trigger a malignant transformation of epithelial cells only by targeting the stroma in which these cells belong. If N-nitroso-methyl urea is administered directly on the epithelial cells, no tumor transformation is elicited [100]. Small modifications in the composition and stiffness of the extracellular matrix are sufficient to modify the regulatory activity of the cell cycle and consequently can inhibit or promote cell proliferation accordingly [101,102,103]. In fact, through its interaction with the cytoskeleton, the microenvironment is able to modulate the transcription of genes and activate or inhibit the various associated molecular pathways [104,105,106,107]. Moreover, changes in microenvironment composition and structure are often associated with the development of fibrosis, the formation of intricate collagen networks, and even tissue stiffening. All of these processes increase the risk of developing cancer [108,109]. Contrarily, a physiological microenvironment is able to favor the processes of apoptosis [110,111] so much that it is possible to induce a reversion of the tumor phenotype by re-normalizing the characteristics of the extracellular matrix [112]. More generally, physical as well as biochemical anomalies in the microenvironment can act by exerting a pro-tumoral action, thus acting as a true oncogenic, “causative” factor [113,114].

Furthermore, as discussed later, oncogenic mutations are also present in normal tissues. This can be interpreted as a clue to the existence of mechanisms—expressed by the “normal” microenvironment—that prevent the malign expression of mutated genes in the microenvironment of healthy tissues [115]. Indeed, the microenvironment can exert a double action: the inhibition of tumors, even in the presence of oncogenes, and the promotion of tumors, even in the absence of gene mutations [113].

However, a full understanding of the dynamics of biological organization requires that we look at genes with new eyes in order to grasp the global dynamics of networks, whose behavior is collective and is regulated at a higher order than that of individual genes kept in isolation [116].

### 4.3. The Integration of the Microenvironment within Biological Models

In consideration of the above, a reliable appreciation of gene activity should be investigated within a complex system that is capable of integrating the characteristics and functions of the microenvironment, the morphogenetic fields, and the entire biological organization [117]. Consequently, it would be possible to understand the phenotypic characteristics, as derived from a complex interaction between the cells and the microenvironment, rather than just a linear, hierarchical correlation between them as posited by the reductionist approach [118].

Denis Noble has thoroughly investigated the generation and propagation of the rhythm of the heart by means of a mathematical model. This is a paradigmatic example of the approach to which we refer. Nobles’ work required a multi-scale approach that included the tissue structure and macroscopic anatomy of the heart, without which the model could not have worked. Functionalities emerge from the interaction of genes, proteins and all the cascades of signals that develop within the microenvironment [119].

Here, the very concept of “genetic information” is questioned [120]. The reductionist framework seems misled by the concept of information as developed in computer science, where a clear meaning of biological information is missing along with the observables that should be taken into consideration [121]. Thereby, the hypothesis that the genes determine the entire biological organization in every detail by “controlling” the flux of “biological information” is becoming weaker and weaker [122].

As mentioned, the reductionist approach focuses on entities, while the systemic/organicist approach focuses on the dynamic relationships among entities. While the reductionist approach has proven to be extremely effective in investigating certain aspects of biology and in developing therapeutic solutions, it struggles when dealing with more complex issues, such as tumor reversion or embryogenesis.

## 5. The Systemic Approach

The word system derives from the Greek verb, *synistanai* (*συνίστημι*), which means to put together, to organize. By system, therefore, we generally mean an “organized whole”, an aggregate of parts that depend on each other according to fixed laws and rules and have the same goal. We may describe a biological system as a network of integrated components that can feature nonlinear dynamics [123]. The organizational structure of the living world seems to obey different hierarchical levels. These range from the subatomic level to the entire ecosystem, in=cluding cells, tissues, and organs. Emerging laws that do not appear at the lower levels of organization simultaneously characterize and govern each of these levels [124].

The investigation of a complex biological system requires the following: (a) understanding how its components relate and integrate into increasingly larger and more complex organizational structures; (b) recognizing the correlations between local processes and the global structure at different organizational levels; (c) investigating how the laws that occur at the organic level can influence the behavior and organization of the lower levels (bottom-up and top-down causation); and (d) studying biological homeostasis, i.e., the way all of the different parts contribute to the robustness of the organic properties. In other words, the space between molecules and life needs to be studied [125].

In an attempt to investigate these issues, two different approaches to the application of systems science in biology have been developed: a purely computational one, based on data and statistical analysis, and a more theoretical one aimed at identifying principles that drive the biological organization. Rather than oppose, these approaches complement each other [126].

What is known as the pragmatic approach focuses on molecules to describe all interactions that occur at this level by means of mathematical and biochemical models. This is the case of the various omics sciences [127]. The theoretical approach argues that it is necessary to rethink the study of biology from both a speculative and a methodological point of view.

Here, we present the contribution of this perspective in the study of tumor reversion processes. We will, therefore, introduce some concepts borrowed from organicism, useful in modeling tumor reversion processes.

### 5.1. Limits of the Pragmatic Approach

One of the main critical issues with the pragmatic approach concerns data production and management. In an effort to study the complex interactions that occur at the lower biological levels, different analytical methods are employed. These include gene expression patterns, microarrays, and all the “omics” technologies, for example, metabolomics and proteomics [128]. The pragmatic approach aims to integrate all data coming from molecular biology within complex computational and mathematical network models [129,130].

There are two main limitations with this approach. First, how can data produced at the molecular level be correlated so that there is a biological meaning for higher organizational levels? Second, how can the conditioning of the epistemic premises in the very process of data collection, selection, and modeling be decided [131].

In fact, data collection is not a purely empirical and neutral activity. Science does not collect data randomly. Rather, it does so through experiments. These are designed and conducted to identify the parameters that are thought to be relevant. Inevitably, the implicit epistemic premises of the experimental model influence the choice of the parameters, hence data. The latter are no longer neutral with respect to theory [132]. The possibility of telling or even perceiving certain facts, data, and objects depends on the point of view of the observer [133]. Privileging the molecular level as causal is an example of how epistemic premises condition the criteria of data collection and the processes of scientific research.

Systems biology’s pragmatic approach takes shape from the belief that causal relationships can be deduced from a mere process of data collection and processing. Big data science is a form of technology-based empiricism. It implicitly affirms the primacy of inductive reasoning and has inspired the idea that future automated data mining leads directly to new discoveries. However, more data do not necessarily generate more knowledge [133,134]. In several cases, it has been found that many correlations observed in certain data sets were spurious and did not indicate a real interdependence [135].

The enormous amount of data produced by the Human Genome Project failed to deliver any of the expected knowledge shifts. This testifies that data alone are insufficient for understanding biological processes [53]. The huge quantity of information accumulated was not only unable to clarify certain phenomena but also increased the distance between data and the comprehension of the organizing principles of biology [136,137].

It does not matter how intense and well-performing the computational activity is. It can never replace a theory for giving laboratory data a biological meaning.

Theory as a guide to experimental design is, therefore, crucial for efficient data collection, as well as producing reliable predictive models and conceptual knowledge [85,138].

According to the reductionist approach, biological information linearly flows from DNA to proteins until the phenotypic expression [127]. This makes any proper investigation into tumor reversion impossible within the SMT framework, since a transformation of tumor phenotype without accompanying changes in specific gene mutations is inconceivable.

The theoretical approach, instead, offers new conceptual tools and even a different lexicon. These include complexity, organizational structures, multilevel organization, non-linear dynamics, network modeling, multi-scale biophysical constraints, and other incomputable aspects of the living world [139]. This allows for new experimental models to highlight and measure relevant observables and better understand the dynamics of tumor reversion. In this sense, it is necessary to clarify some concepts of complex systems that may play roles as descriptors and methodological tools.

### 5.2. From Entities to Relations

In order to understand a biological organism, it is necessary to look beyond the intrinsic properties of the individual entities and consider the relational dynamics that exist between them [131,140]. One cannot define what biological entities can or cannot do only by investigating their internal properties. The organism’s behavior rather depends on the integrated set of interactions between its different elements [140].

Isolated entities may not show the same behavioral properties in different contexts. For example, cell lines cultured in vitro may produce data and information inconsistent with the organic context of in vivo studies [141].

Indeed, the functional properties of a biological organism are not “intrinsically” inherent in its individual components. Rather, they emerge because of a specific organization among these various parts. This organized structure features properties that do not directly come from its components. Instead, the overall configuration exerts a “binding” and regulatory action on the components themselves [142]. This introduces the distinction between the intrinsic properties of individual entities, i.e., the properties that entities have by virtue of what they are, and relational (“emergent”) properties, i.e., the properties that entities have as a consequence of the way in which they interact with each other and with other environmental structures [140].

Even in the inorganic world, molecular interactions occur within complex matrices including water [143], electromagnetic fields [144], and gravitational fields [145]. All of these elements contribute to a single background field that guides and constrains chemical reactions.

A paradigmatic example is the different organizational models of carbon atoms that characterize two very different materials, i.e., graphite and diamond. It is clear that the property of hardness does not depend on the specific entities of carbon atoms but on how these are organized, that is, how they relate to each other. Even the property of acidity, which corresponds to the ease with which a substance in water releases H+ protons, depends on relations: no compound can be defined as acidic or basic per se. It can behave as an acid or a base depending on the context, that is, depending on the other substances in the solution, which may be more or less prone to release H+ protons.

In the same way, genes for cells and cells for tissues can modify the properties of higher systems based on how they are organized. The opposite is also true in biology, i.e., higher-level context and dynamics can affect the activity of genes and cells. From this perspective, it is agreed that causal processes proceed both from the microscopic world toward the macroscopic and vice versa [146].

Thus, when separated from their neighbors, cells lose most of their functional and structural attributes. A sort of causality reverberates from higher to lower levels: macromolecules, metabolites, genes, and proteins are all intimately linked to each other. They form an integrated system that changes according to the stimuli coming from the higher levels.

This approach requires a style of systemic reasoning that does not consider observables as autonomous entities within the system but focuses on the relationships between them.

Explanatory models should, therefore, search for the appropriate biological observables where target phenomena and their meaningful correlations occur [147,148].

As Noble showed, it is impossible to develop explanatory models of the functioning of the heart or any organ by exclusively studying its genetic level [119]. There, stochastic fluctuations in gene expression generate disorder [149]. However, leveling up makes the collective coherence of the non-linear dynamics of the lower levels emerge: the same processes that appear chaotic on lower scales give rise to ordered structures at the mesoscopic level. In principle, the same phenomenon can be studied at different levels: from the atomic one to the cellular one. A system, therefore, appears different according to the various levels of magnification; all of the levels concur in the form and functionality of the system, but the mesoscopic level should be the privileged level of observation. In fact, it is there that the most scientifically relevant phenomena can be observed [125,141,150].

In short, the difference between reductionism and the systemic approach lies in this: the reductionist approach considers it theoretically possible to derive all of the properties of an organism from its components. The systemic approach considers this impossible because organic forms and functions emerge gradually from the non-linear interaction between different sub-structures.

It is interesting what Denis Noble points out: evolutionary processes rarely act on single cells or distinct species. Rather, they affect complex multi-scale systems and the non-linear way that components interconnect [151].

### 5.3. Bottom-Up and Top-Down Causation

Unlike a machine, where each function is directly deducible from the characteristics of its components, living organisms show “emergent” properties that cannot be deduced from fundamental laws or single parts [150]. Each level in biology is governed by emerging laws that do not appear at the lower levels.

It is key to understand how these emerging properties can influence the lower levels with a top-down causation, and how the lower levels can determine certain higher-level behaviors via a bottom-up causation [152,153].

In fact, living organisms are hierarchically organized so that the dynamics that occur at the lower scales integrate with the constraints that come from the higher levels. This determines the rules of functioning and adaptation [154,155].

The systemic approach to biology aims to understand how causality operates and functional processes integrate on the different levels [127]. Specifically, it seeks to identify the levels of the system in which the most relevant dynamics take place. In this sense, the mesoscopic level is where biological dynamics acquire greater coherence in terms of causal correlations. There, the effects of constraints coming from the higher scales harmonize with the stochastic dynamics of microscopic scales. This integration produces emerging properties [156]. Order within living systems is mainly imposed by higher levels in the form of general constraints and forces such as electromagnetic, gravitational, and mechanically transduced forces dependent on cells and tissues [148,157].

Explaining cancer in genetic terms does not mean that cancer is a genetic phenomenon. There is an explanatory asymmetry between the level at which a phenomenon is explained and the terms in which it is explained [71]. If it is true that every biological phenomenon is molecular, it is also true that no phenomenon is just molecular. An approach based only on molecules only ignores the relevance of morphological forms and morphogenesis. In this sense, cancer can be seen as a developmental phenomenon emerging at the tissue level [158].

Here, the morphogenetic field takes on a central role in determining the constraints and dynamics by which living systems organize and adapt. The laws of motion channel the possible movements of the planets. Likewise, the morphogenetic fields can determine biological processes [125,159,160].

### 5.4. The Morphogenetic Field Concept

The “morphogenetic field” concept arises from the first speculations about the laws of form developed by D’Arcy Thompson in the early 1900s. This author hypothesized that it was possible to understand the laws of biological development through a mathematical modeling of different living forms and their mutation [161]. The morphogenetic field can be described as the result of the integration of biochemical and biophysical forces. Under this field, the various degrees of freedom of the biological components and processes are bound to an “ordered pattern” that integrates the functions of its parts into the integral activity of the whole system [162].

For a long time, the morphogenetic field concept was used as an analogy, but since the 1980s it has been a fundamental concept for the study of developmental biology [163,164]. During early developmental steps, the fertilized egg draws the topological information necessary for its development mostly from its microenvironment. A fine program guides the progressive differentiation of the cells and their space–time organization according to three growth axes (dorsoventral, right–left, and craniocaudal). Such a program depends on the interaction of the cells with their field [165]. This is a very critical point, and consequently, morphogenetic processes have come of age, becoming a field of useful theoretical and methodological tools [166,167].

Cells lose many of their differentiated functional characteristics when isolated and placed in a culture medium. This shows that cellular specialization depends on the context, i.e., the morphogenetic field [168]. Cells are not a homogeneous colloidal soup in which processes occur following the classical laws of diffusion and kinetics. Rather, their highly organized environments obey laws that go well beyond those of Newtonian fluids [169]. For this reason, it is impossible for the genetic code to dictate every detail of a biological form [122].

The morphogenetic field binds cells to a specific dynamic adaptation to external stimuli, such as shear or traction forces, compression, hydrostatic pressure, and even electromagnetism. Cellular response occurs through cytoskeletal changes in shape and behavior. These modifications can, in turn, exert an influence on both their microenvironment (mechano-reciprocity) and gene expression in a self-regulating mechanism that guarantees the cell’s homeostasis [170,171,172]. In this framework, what matters is the set of reciprocal relationships rather than the behavior of individual isolated elements. Every single entity behaves according to the rules and schemes imposed by the system, like an orchestra that plays following the higher organizational level of the score.

The cellular microenvironment and its morphogenetic field can play a decisive role in the development or regression of cancer. Their effects on tissue organization and cellular interaction processes can activate or block mechanisms such as apoptosis, cell proliferation, and even migration [173,174].

This framework allows for shifting the focus from “local”, lower scale systems to more complex global networks. This can be seen as an expansion of Waddington’s early metaphorical conceptualization of the morphogenetic field, i.e., the “epigenetic landscape”, where a biological system can move toward different states depending on the topology. The landscape can change in response to genetic, physical, and environmental signals. Even slight and gradual variations in a single parameter can affect non-linear processes and determine significant phenotype changes [175].

Waddington’s landscape has been conceptualized as “phase space” by the theory of dynamic systems. The resulting mathematical formalization can represent any state of the system, independent of its observable parameters [176,177].

A unified model of multilevel complex dynamical systems consisting of interacting molecules, physical signals and intra- and extracellular structures was proposed under the name of interactome [178]. The interactome is a graph of all of the complex networks of molecules, proteins, genes, physical factors, and any other element constituting a living organism. The interactome of *Saccharomyces cerevisiae* yeast, for example—the most complete to date—lists over 20,000 interactions between proteins [179] and over 170,000 interactions between genes. This model made it possible to create functional maps of cellular processes where genes with similar functions are grouped together. The map allows for observing the processes of genetic interaction from different levels. The interactome contributed a lot of new information on network dynamics and produced several relevant observations. For example, scientists found that the negative (inhibitory) interactions are much more numerous than the positive (stimulatory) ones. They also understood that the genes with the greatest number of connections are the most vital for the network. In fact, their mutation or perturbation may generate lethal effects on the entire network [180].

Nonetheless, such models still have several limitations. Many elements such as bond strength, sensitivity to signals, microenvironment factors, specific physiological states of the cell, and electromagnetic factors, to name a few, can play a crucial role in biological processes but are not integrated within the interactome [181].

A biological organism, whatever its level of organization, can, therefore, be described from the point of view of complex networks. This allows the natural convergence of micro-, meso-, and macroscopic data, i.e., the harmonization of the individual nodes, the cluster of nodes, and the entire network [182].

### 5.5. Conceptual Tools for Describing a Biological Network

The interpretative framework based on the theory of systems and complex networks leads to new experimental questions and observables. It offers new conceptual tools to interpret phenomena that were previously difficult to explain, such as reversion tumor.

As mentioned, a biological network can be described by means of the characteristics of nodes and links at different scales.

A node is any element with a relevant role in the system, for example, a gene, a protein, a molecule, or a cell. The key element of a node is called “degree”, i.e., the number of connections. A network consists of a series of nodes linked to each other via correlation factors, physical interactions, or spatial proximity. A node with many connections is called a “hub” [183]. At an upper scale, it is possible to evaluate the “connectivity”, that is, the density of connections in the network. On the other hand, “module” indicates a group of nodes characterized by a high density of connections. In turn, modules can be connected to each other. The mathematical structure that describes this network organization is a graph [147].

Within a network, the dynamics of each component depend on the simultaneous dynamics of the other components. The result is a correlated behavior as if from a single entity. These collective dynamics are the basis of all of the adaptive and evolutionary movements of the system and allow for a continuous adaptation that preserves the internal coherence of the organism.

The main types of network organization are exponential and scale-free. An exponential network is largely homogeneous and has about the same number of links per node. Its nodes are very unlikely to feature many links. On the contrary, scale-free networks are not homogeneous—most of their nodes have few connections, while some have a large number of connections. Scale-free networks characterize biological organisms, where the laws emerging at each level cannot stem directly from the laws governing the lower levels [184]. Further, the dynamics and characteristics that emerge on higher scales can influence the lower levels, for example, by providing constraints to cell behavior [150]. This significantly limits the number of possible conformations of the system [185,186], i.e., the number of network conformations. The dynamics of these systems are non-linear, that is, the extent of the effects and their variations is not proportional to the causes and their variations. Accordingly, small lower-scale variations can determine large modifications at the upper scale [187]. Unlike linear systems, nonlinear dynamic systems can appear chaotic and unpredictable. Most natural systems are nonlinear [188].

**Figure 1:**
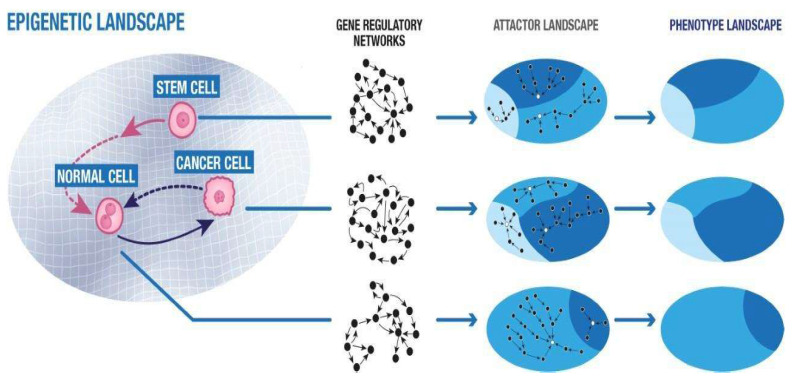
The Waddington epigenetic landscape is modeled as a field with hills and valleys. To each hill corresponds a metastable state, to each valley a stable state. Each stable state can be associated with a specific gene regulatory network that corresponds to a specific attractor landscape. The result for each attractor landscape is a corresponding phenotype such as somatic cell, cancer cell or stem cell.

A system switches from a linear to a non-linear regime when one or more parameters of its state fluctuate above a certain threshold value. Beyond this threshold, the system reaches a bifurcation point and accesses the possibility of reorganizing itself in a new stable conformation. This process is known as symmetry breaking. It gives the system the characteristic of multistability, i.e., the possibility of stabilizing in different ways and, therefore, adapting. The various stable conformations of the system are called “attractors”, which mathematically represent the solutions of the set of equations that describe the system. In terms of Waddington’s landscape metaphor, the attractors are stable states, i.e., “valleys”. Metastable and unstable states, instead, are “hills” (Figure 1) [189].

The system can change state moving toward different attractors thanks to the bifurcation points that lead to a break in symmetry, that is, a metastable state that favors a sort of phase transition. The symmetry break gives the system a historical dimension, a sort of memory of an event that occurs at a critical point and will influence the next evolution. This leads to relevant consequences studied in depth by thermodynamics [190].

From a thermodynamic perspective, a living organism is described as an open system that is far from equilibrium. It is characterized by dissipative structures that self-organize through fluctuations between stable and unstable states [191]. Each fluctuation corresponds to a bifurcation following the system’s state change. These approaches have contributed to the development of non-equilibrium thermodynamics and have been a prelude to systems biology. In fact, they have provided further tools for the analysis and modeling of biological processes [192].

## 6. Interpreting Tumor Reversion Processes within the Systemic Framework

The systemic perspective offers models, methodological tools, and a lexicon that can contribute to a more complete interpretation of tumor reversion processes.

From this perspective, attention shifts from entities to relationships, from genes to the cellular context, and from the characteristics of DNA to the processes that guide the entire organization of cells in higher-level structures. We no longer interpret the tumor as an entity in itself but rather a pathology of biological organization.

The main issue relates to the concept of identity: what is cancer? Such a question recalls the famous essay by Erwin Schrödinger, *What Is Life?* This famous physicist investigated what allows living organisms to remain the same over time and, therefore, their identity principle [193].

Clearly, DNA represents the element of continuity that, from cell division to cell division, reproduces copies of itself and maintains the unaltered, genetic “identity” of the cell. In this case, genetic identity refers to the precise sequence of purine and pyrimidine bases (ATCG).

However, each cell expresses only some genes of the entire DNA. For this reason, different cell types are distinguished at the histological level: hepatocyte, neuron, cardiomyocyte, mesenchymal stem cell, etc., as their identity is defined “phenotypically”.

In fact, the well-known *hallmarks of cancer* are criteria that do not allow us to affirm that the tumor cell owns a proper ontology, identified by a well-recognizable genotype; rather, the identification is based upon structural and behavioral traits [194,195]. Moreover, during their life cycle, cells can change their behavior and phenotypic expression in reaction to external stimuli: the wound healing processes in which involved cells acquire a behavior that is very similar to that of cancer for a defined period are an emblematic case. Biological identity is something that goes beyond both the concepts of genotype and phenotype.

Similarly, tumor reversion represents a case of modification in phenotypic expression: the same genes, even if mutated, change the way they express and, consequently, the features and behavior of the cell. In this sense, research on tumor reversion shifts attention to the relationship between genotype and phenotype and aims to study the dynamics that direct the process of phenotypic expression.

### 6.1. Genotype and Phenotype

Increasing evidence shows that the idea of a linear correlation between genotype and phenotype does not correspond to the reality of biological dynamics [196]. For a long time, it had been thought that each gene encoded a specific protein or characteristic of the cell. This concept entered the collective imagination so much so that even today we speak of a “green eyes gene”, “height gene”, or even, entering the psychological domain, an “empathy gene” or “jealousy gene”. Although these expressions are more attributable to popular simplifications, the basic concept of a linear correlation between genotype and phenotype remains. This is also one of the fundamental elements of SMT, in which proto-oncogenes and tumor suppressor genes are considered linearly correlated to tumors. The reality has proven to be much more complex: not only do several phenotypes correspond to a genotype, but also, in some cases, the opposite is true, i.e., a phenotype can be determined by different genotypes. These data indicate that there is a non-unique genotype–phenotype relationship, suggesting that the “robustness” of the phenotypic state cannot be attributed linearly (only) to gene configuration [197].

These processes can, in fact, be described using concepts such as network, network state, phase space, attractor, and epigenetic landscape. The phenotype can, therefore, be interpreted as a specific functional state of the cell that results from the expression of a well-defined combination of genes [41]. These concepts should be considered together with factors such as cell–microenvironment interaction and both macro- and microscopic system constraints. In other words, it is a matter of studying these processes from a topological point of view, taking the morphogenetic fields into account.

Phenotypic changes are determined by one or more perturbations that destabilize the entire system. These perturbations cancel the action of the attractor corresponding to a specific state of phenotypic expression—a “valley” [198] or, in embryology terms, a morphogenetic field [164]—and trigger a non-linear transition. This means that the system does not necessarily pass from A to B but is in a multistable state—a bifurcation that can lead to a state B or a state C attractor [41]. The stochastic nature of these processes can be appreciated when the attention shifts from a single cell to a population of cells. The population will react by presenting a series of new stable states or phenotypes that are stochastically distributed [199].

In addition, it should be considered that the phenotype characteristics are not determined exclusively by the activation or deactivation processes of gene expression, but also by processes of non-genetic plasticity that involve the dynamics of post-transcriptional regulation. This has also been observed in cancer cells [200,201].

All of these dynamics contribute to determining the functional state of individual cells, their organizational architecture, and, consequently, the characteristics from which functional tissues derive.

Therefore, it is theoretically possible to identify a discrete and finite number of classes of attractors that correspond to the configurations allowed by their genetic and biophysical constraints. The set of these correlation constraints and environmental factors drastically reduces the number of conformations that the system can take by channeling the behavior of the cells [185,186,202].

In order to provide relevant information, this type of model requires a huge amount of data [185]. For this purpose, it relies on techniques such as metabolomics, proteomics, and single-cell gene expression [203,204] for in silico simulations.

An interesting example is the gene regulatory network (GNR). Genes are not independent entities but belong to complex networks known as “gene regulatory networks”. Here, genes influence and bind to each other, further limiting the possible combinations of gene expression [91].

The GRN structure represents a constraint for the genes and a sort of “background field” that coordinates its functioning. Within the metaphor of the Waddington landscape, each valley corresponds to a stable state of phenotypic expression that derives from a precise configuration of the GNR [205]. When a cell, following a perturbation, changes state and, therefore, its phenotype, what changes is not the way in which the different genes are connected to each other, that is, the GRN, but the level of expression of the various genes. Because of such an interlinked and coordinated process, a single cell’s change of state cannot be triggered by the change in expression of a single gene. For that, it takes many genes to do so, and only according to a specific set of combinations. Such a configuration can be defined as an attractor and corresponds to the new state of the cell. This information has been made possible by new techniques, such as those of single cell transcriptomics [206]. This allows for analyzing gene expression at the level of single cells and, for example, contributes to the study of tumor heterogeneity, i.e., the presence of different types of cells within the same tumor mass.

Since the stability of cell functional states also depends on external signals [207], this approach risks having several limitations.

We are now able to interpret cancer as a particular state of the cell that evolves over time within the “landscape model”. This transition from one state to another is fundamentally determined by two variables: the external signals and constraints, and the internal adaptive response of the cell.

Accordingly, cancer should be interpreted as an epiphenomenon that emerges from the disintegration of the cell–microenvironment system. Hence, it is essential to identify suitable indicators to highlight the state of system integration, as well as the correct level of observation.

The consequent therapeutic strategy should, therefore, no longer try to eliminate tumor cells. Rather, it should aim to induce a benign modification of the phenotype. Tumor reversion research points out that this phenotypic change can be induced through specific biological signals. These modify the microenvironment or, more generally, the morphogenetic field, and channel the cell toward new stable states.

### 6.2. The Systemic View of Pathology

Disease can be interpreted as a non-linear process that is subject to various subsystems, different attractors, and their multistability, resilience, and robustness. The redundancy of alternative pathways within the network allows the expression of the same phenotype through different attractors [94].

The landscape model may also describe pathological states. Here, stable states (“valleys”) represent the physiological states of health, while unstable states (“hills”) represent the unstable processes that determine the onset of a pathology. When an external agent (a pathogen) disturbs the system, then the system tends to move away from its attractor and signals the fluctuation of certain critical parameters [208]. The perturbation can lead the system to overcome the “energetic” boundary of the attractor, thus opening up the possibility of moving toward new attractors and, therefore, new stable states. This transition from one attractor to another can take place gradually or abruptly depending on the type of pathological process. Based on the type of progression of the pathophysiological state along the landscape, the process can be classified into three different stages: normality, pre-disease, and disease. Notice that the three are steady and stable states [209]. These three distinct states present a series of intermediate critical stages, which are highly unstable. This means that the pathological process can take radically different directions: either toward the progression of the disease or toward recovery.

By modeling the disease process in this way, the sudden deterioration of a state can be seen as a phase of transition occurring at a bifurcation point [210].

The various critical stages, as well as the pre-illness stage, therefore, correspond to bifurcation points. Here, the fate of the system depends on a set of internal and external conditions, which can guide the process toward very different destinies. Recognizing such critical points by building a dynamic network will likely help to understand the logic of the process [211]. Biomarkers such as metabolites can signal an impending bifurcation or a sudden deterioration before the critical transition occurs. These early warning signs can help plan an appropriate management of the disease [212].

Indeed, the current challenge is to move from molecular parameters linked to specific targets toward the system parameters of the pathological process and its state. This approach is partly developed through the study of metabolomics [213,214], since fluctuations in metabolites usually amplify the subtle modulation of the genome and proteome networks, thus representing a more sensitive criterion for capturing changes in the dynamics of complex systems [215,216]. In this sense, it can be said that metabolomics offers the best representation of the phenotype to date [204].

In conclusion, it is impossible to deterministically predict the patho-phenotype of even a monogenic disease [217].

We can read the pathological process as a complex and multilevel network of non-linear interactions between the various components of the organism. In fact, a complex network of non-linear interactions between stroma, extracellular matrix (ECM), and epithelium guides the development of tissues [218]. The relevance of cell–tissue relationships suggests that tumors may be tissue-originating pathologies [219,220]. Evidence suggests that cancer develops as a consequence of the interruption of interaction between cells and their microenvironment. This provides for unexpected and complex changes in cell morphology, signaling pathways, and genomic functions [100]. The causes of this condition should no longer be sought exclusively at the lower levels of the organization, but also at the higher levels where the phenomenon appears [182,221].

### 6.3. Tumor Reversion as a Reorganization/Transition Process

Multilevel organization is an intrinsic characteristic of living organisms. The terms “organism” and “organization” share the same roots, from Greek ὄργανον (“tool”, “what exercises a function”) and ἔργον (“work”, “action”). Both terms refer to an energy aimed at a specific function. In other words, organism and organization are both structured systems for channeling energy in such a way as to perform a specific function.

If a tumor is a “pathology of biological organization”, then network science offers the best language for defining it. Accordingly, we want to propose the use of the term “reorganization”, which refers to a transformation process directed toward a more organized state than the starting one. The empirical term “reversion” suggests a backwards process of the cells. The term “differentiation” does not take into account the spontaneous processes of apoptosis. The term “regression” says nothing about the typology of its cytotoxic, spontaneous, or differentiative processes. On the contrary, reorganization offers a more complete and centered perspective within the conceptual framework of the systemic approach. It does not refer exclusively to a single cell. Rather, it directs attention to the organizational aspect that can be both internal and external to the cell because there is a network of cells in the environment.

We propose the introduction of the expression, “process of cellular reorganization”, to indicate a biological-state transition process.

## 7. Conclusions: Challenges and the Way to Move Forward

**Figure 2:**
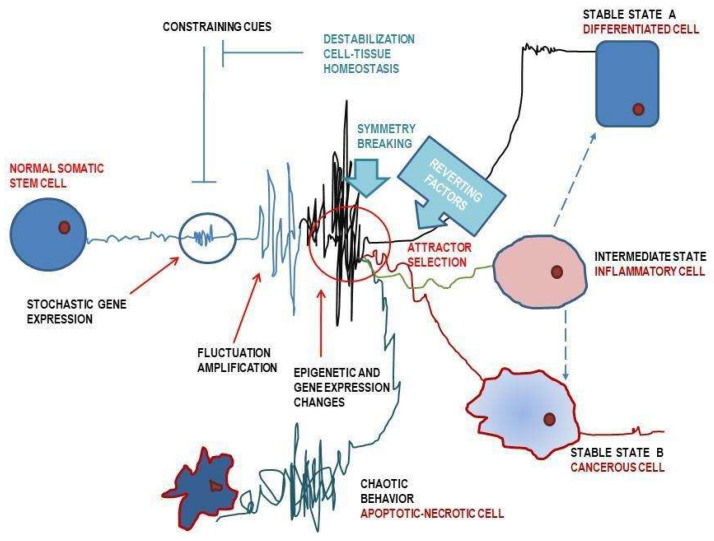
Normal and cancerous phenotypic reversion. Somatic and stem cells undergo critical phenotypic transitions during physiologic (cell repair, mitosis) and adaptive (epithelial–mesenchymal transition) processes, as well as in response to perturbations that destabilize their equilibrium in a previously stable basin of attraction. Once the system’s resilience exceeds a certain threshold, the system experiences an increase in the fluctuation of a few, critical parameters. At this point, several molecular and biophysical factors—namely those affecting stability of cell-to-cell adhesion and microenvironment architecture—can efficiently drive the system toward a new, stable and differentiated phenotype. However, the coexistence of an inflammatory condition, deregulated microenvironment homeostasis, or other unknown factors can direct the transition towards different outcomes (inflammatory phenotype, cancer). By analogy, even a cancerous cell, when challenged by perturbing cues, can be displaced from its stable state and re-enter into a differentiating pathway. If proper constraints and differentiating factors are in place, the overall process can lead to a “reversion” of the cancerous phenotype by following an “inverse” path.

Several studies have shown that numerous molecular and biophysical factors—namely those obtained from eggs/embryonic cells/microenvironments—can efficiently promote the reversion of the cancerous phenotype toward a “normal” physiologic condition [222]. Noticeably, those results highlighted that tumor reversion can be accomplished when proper cell-to-cell and cell-substrate adhesion structures are restored. There is no doubt that a critical role is sustained by the reconstitution of E-cadherin-based junctions associated with a rewiring of the overall cytoskeleton (CSK) structure. Changes in cell architecture are instrumental in modifying tissue properties and cell responsiveness to mechanical stimuli. Furthermore, changes in CSK involve epigenetic and post-translational modifications that ultimately can significantly antagonize the malignant phenotype (Figure 2). Namely, changes in the mechano-transduction apparatus can have a profound impact even on the malignant behavior of cancer stem cells that can lose stemness and malignant-related properties when challenged by different stiffness conditions [223]. However, these studies are still in their infancy, as a number of questions have been left aside [224]. What should be a reliable model to vindicate these preliminary results? How to validate in vivo—both in animal studies and in clinical trials—those promising insights? Specifically, a “strategy” to trigger the reversion should include the preliminary destabilization of the cancer state that displays an appreciable resilience to a wide range of perturbations. Indeed, only after the cell has entered a condition in which fluctuation in gene expression approaches a threshold value, “reverting” factors can display their powerful effects in driving the system toward a different phenotypic configuration. Cancer cells reach a critical transition state during mitotic/regenerative processes, although their stability can be proficiently perturbed by several factors, including drugs and molecules affecting the microenvironment homeostasis. Indeed, tissue regeneration is a delicate procedure; such a procedure is closely related to the possibility of neoplastic transformation when proper constraints and directional cues are not activated to address the transition toward a physiologic outcome. Thereby, a “reverting” treatment plan should include a proper roadmap in which a destabilization “procedure”—eventually triggered by anti-cancer drugs—should be followed by the addition of reverting drugs. Furthermore, such a protocol must be tested in animals to ascertain the treatment effectiveness in vivo. Promising studies have been already published, but the evidence is still scarce. Undoubtedly, there is a long way. But we must begin with the first step.

## Figures and Tables

**Table 1:** This table organizes the main experimental works in which a “tumor reversion process” has been observed or induced. Each work is presented highlighting the tumor model, the experimental methodology, the results and some relevant comments.

Tumor Model	Experimental Methodology	Results	Comments	Ref.
Ovarian teratocarcinoma	Clinical observation	Spontaneous regression, differentiation of cancer cells into normal tissue		Askanazy, 1907 [1]
Plant teratoma clonal cells	Succession of grafts on healthy tobacco plant	Disappearance of the teratoma and plant generation with seeds capable of giving life to a new plant	The results led the author to introduce the concept of pluripotentiality of cancer cells	Braun, 1959 [16]
Murine embryonic tumor cells	Transplantation on mice	Differentiation of cells	Hypothesis on the role of the tissue context in determining the fate of cancer cells	Pierce, 1961 [4]
Hamster sarcoma cells (induced by Rous sarcoma virus)	Succession of cell cultures and re-platings	Transformation of 19% of cells, which return to orienting themselves in an orderly manner, as in healthy tissues	The author uses the term “reversion” to describe results	Macpherson, 1965 [17]
Murine testicular teratocarcinoma cells (black mice)	Injection into murine blastocyst implanted in albino female mice	Development of healthy mice	One of the mice had black tufts, characteristic traits of the genome of teratocarcinoma cells	Brinster, 1974 [5]
Embryonic carcinoma cells from black mice	Blastocyst injection implanted in brown female mice	Normal fetal development; normal newborn mice feature hybrid traits between black and brown mice	Authors use the term “reversion” to describe their findings	Mintz and Illmensee, 1975 [6]
Lucke renal tumor cells (tumor of viral origin)	Planting on regenerative salamander limbs	Stopping of tumor growth and subsequent differentiation of cells	Failed to determine whether the differentiated cells came from cancer cells or healthy tissue	Rose and Wa llingford, 1948 [18,19]
Spontaneous tumors in animals	Observations on the rate of onset	Reduced occurrence of tumors in animals with high regenerative capacities		Gersch, 1951 [20]
Triton-induced epithelial tumors	Monitoring the spontaneous evolution of tumors	Tendency to tumor regression in anatomical areas with high regenerative potential	Results confirmed by histological analysis	Seilern-Aspangand Kratochwil, 1962 [21]
Liver cancer cells (marked with dye)	Injected into liver tissue	Reduction of malignancy and, in some cases, differentiation of cancer cells	Use of the term “partial reversion” to describe the phenotypic change of cells	Coleman, 1993 [22]
Neuroblastoma cells	Injected into 8 ½ day old murine blastocyst	Differentiation of tumor cells		Podesta, 1984 [23]
Leukemia cells	Injected into 10-day-old murine blastocyst	Correct hematopoietic maturation		Gootwine, 1982 [24]
Rous sarcoma virus	Injected into chicken embryos	No tumor development	If the virus is injected into adult chickens, then sarcoma develops	Dolberg and Bissell, 1984 [7]
Mouse melanoma cells	Implanted into embryos in murine uterus	Cell differentiation and normal embryonic development	Differentiation occurs when cells are implanted into a 14-day embryo	Gerschenson, 1986 [9]
Murine breast adenocarcinoma cells	Exposure to diffusible substances of murine embryonic mesenchyme	Differentiation of tumor cells		DeCosse, 1973 [25]
Primary murine lung cancer	Administration (in vivo) of homogenates of pregnant murine uteri	Suppression of tumor development		Biava, 1988 [26]
Glioblastoma, melanoma, renal adenocarcinoma, breast cancer, and lymphoblastic leukemia cells	Exposure to embryonic extracts of zebrafish taken before gastrulation	Reduction of cell proliferation rates		Biava, 2001; 2002 [27,28]
Human melanoma cells	Implanted in zebrafish embryos in the early stages of development	Suppression of malignant tumor phenotype and birth of healthy fish		Lee, Hendrix, 2005 [10]
Leukemia cells	Retinoic acid administration	Differentiation of leukemia cells into granulocytes, subsequently digested by macrophages	Today, acute promyelocytic leukemia is treated in most cases with differential treatments based on retinoic acid	Breitman, 1980 [29]
Various types of human tumors	Clinical remarks	Spontaneous regressions of tumors	Several cases of spontaneous regression of tumors have been analyzed, confirmed, and classified	Rohdenburg, 1918; Everson and Cole, 1966; Challis and Stam 1990; O’Regan and Hirshberg, 1993; Papac, 1998 [30,31,32,33,34]
Advanced hepatocarcinoma (179 cases)	In vivo administration of extracts of zebrafish embryos	20% of cancer regressions, of which 2.5% total stabilization progression in 16% of cases	Partial or complete disappearance of liver cancer in terminally ill patients	Livraghi, 2005 [35]
Melanoma cells and breast cancer cells	Exposed to embryonic stem cell factors	Reversal of the malignant phenotype and activation of apoptotic processes (nodal signal inhibition was also observed)	If cells are exposed to factors extracted from umbilical cord and bone marrow stem cells, then no phenotypic reversion is observed	Henrix, 2007; Postovit, 2008 [36,37]
Ovarian, prostate, and breast cancer cells	Microenvironmental exposure of human embryonic stem cells	Reversion of malignant phenotype block of cancer cells in phase G1	These results led researchers to hypothesize an inhibitory action on the cell cycle by factors extracted from embryonic stem cells	Giuffrida, 2009 [38]
Melanoma cells	Microenvironmental exposure of human embryonic stem cells	Reversion of malignant phenotype	The study identified some mRNAs involved in these cellular reprogramming processes.	Costa, 2009 [39]
Breast cancer cells	Exposure to salamander, frog, and mouse embryonic cell extracts	Stable reversal of malignant phenotype (confirmed with subsequent implantation of reprogrammed cells in immunosuppressed mice)	Re-expression of some cancer suppressor genes has been observed; mouse embryonic cells did not give results	Allegrucci, 2011 [40]
Breast cancer cells	Exposure to embryonic extracts of zebrafish taken at different times of embryogenesis	Reduction of invasiveness, migration, and proliferation parameters; action on cytoskeleton and TCTP downregulation	An activation method of reversion was identified, implying the down-regulation of TCTP by exposing the cells to a specific embryonic microenvironment composition that corresponds to a specific phase of embryogenesis	Proietti and Bizzarri, 2019 [12]

## Data Availability

Not applicable.

## References

[1] Askanazy M. (1907). Die Teratome nach ihrem Bau, ihrem Verlauf, ihrer Genese und im Vergleich zum experimentellen Teratoid. Verh. Der Dtsch. Pathol. Ges..

[2] Stevens L.C., Little C.C. (1954). Spontaneous testicular teratomas in an inbred strain of mice. Proc. Natl. Acad. Sci. USA.

[3] Pierce G.B., Dixon F.J. (1959). Testicular teratomas. I. Demonstration of teratogenesis by metamorphosis of multipotential cells. Cancer.

[4] Pierce G.B., Verney E.L. (1961). An in vitro and in vivo study of differentiation in teratocarcinomas. Cancer.

[5] Brinster R.L. (1974). The effect of cells transferred into the mouse blastocyst on subsequent development. J. Exp. Med..

[6] Mintz B., Illmensee K. (1975). Normal genetically mosaic mice produced from malignant teratocarcinoma cells. Proc. Natl. Acad. Sci. USA.

[7] Dolberg D.S., Bissell M.J. (1984). Inability of Rous sarcoma virus to cause sarcomas in the avian embryo. Nature.

[8] Pierce G.B., Pantazis C.G., Caldwell J.E., Wells R.S. (1982). Specificity of the control of tumor formation by the blastocyst. Cancer Res..

[9] Gerschenson M., Graves K., Carson S.D., Wells R.S., Pierce G.B. (1986). Regulation of melanoma by the embryonic skin. Proc. Natl. Acad. Sci. USA.

[10] Lee L.M., Seftor E.A., Bonde G., Cornell R.A., Hendrix M.J. (2005). The fate of human malignant melanoma cells transplanted into zebrafish embryos: Assessment of migration and cell division in the absence of tumor formation. Dev. Dyn..

[11] Cucina A., Biava P.M., D’anselmi F., Coluccia P., Conti F., Di Clemente R., Miccheli A., Frati L., Gulino A., Bizzarri M. (2006). Zebrafish embryo proteins induce apoptosis in human colon cancer cells (Caco2). Apoptosis.

[12] Proietti S., Cucina A., Pensotti A., Biava P.M., Minini M., Monti N., Catizone A., Ricci G., Leonetti E., Harrath A.H. (2019). Active Fraction from Embryo Fish Extracts Induces Reversion of the Malignant Invasive Phenotype in Breast Cancer through Down-regulation of TCTP and Modulation of E-cadherin/β-catenin Pathway. Int. J. Mol. Sci..

[13] Lichtenberg F.R. Has Medical Innovation Reduced Cancer Mortality? NBER Working Paper No. w15880. https://ssrn.com/abstract=1586687.

[14] Hanahan D. (2014). Rethinking the war on cancer. Lancet.

[15] Proietti S., Cucina A., Pensotti A., Fuso A., Marchese C., Nicolini A., Bizzarri M. (2020). Tumor reversion and embryo morphogenetic factors. Semin. Cancer Biol..

[16] Braun A.C. (1959). A Demonstration of the Recovery of the Crown-Gall Tumor Cell with the Use of Complex Tumors of Single-Cell Origin. Proc. Natl. Acad. Sci. USA.

[17] Macpherson I. (1965). Reversion in Hamster Cells Transformed by Rous Sarcoma Virus. Science.

[18] Rose S.M., Wallingford H.M. (1948). Transformation of renal tumors of frogs to normal tissues in regenerating limbs of salamanders. Science.

[19] Rose S.M. (1948). Epidermal dedifferentiation during blastema formation in regenerating limbs of Triturus viridescens. J. Exp. Zoöl..

[20] Gersch M. (1951). Zellentartung und Zellwucherung bei wirbellosen Tieren. Arch. Geschwulst-Forsch..

[21] Seilern-Aspang F., Kratochwil K. (1962). Induction and Differentiation of an Epithelial Tumour in the Newt (*Triturus cristatus*). Development.

[22] Coleman W.B., Wennerberg A.E., Smith G.J., Grisham J.W. (1993). Regulation of the differentiation of diploid and some aneuploid rat liver epithelial (stemlike) cells by the hepatic microenvironment. Am. J. Pathol..

[23] Podesta A.N., Mullins J., Pierce G.B., Sells R.S. (1984). The neurula state mouse embryos in control of neuroblastomas. Proc. Natl. Acad. Sci. USA.

[24] Gootwine E., Webb C.G., Sachs L. (1982). Participation of myeloid leukaemia cells injected into embryos in haemato poietic differentiation in adult mice. Nature.

[25] DeCosse J.J., Gossens C.L., Kuzma J.F., Unsworth B.R. (1973). Breast cancer: Induction of differentiation by embryonic tissue. Science.

[26] Biava P.M., Fiorito A., Negro C., Mariani M. (1988). Effects of treatment with embryonic and uterine tissue homogenates on Lewis lung carcinoma development. Cancer Lett..

[27] Biava P.M., Bonsignorio D., Hosha M. (2001). Cell proliferation curves of different human tumor lines after in vitro treatment with Zebrafish embryonic extracts. J. Tumor Marker Oncol..

[28] Biava P.M., Bonsignorio D. (2002). Cancer and cell differentiation: A model to explain malignancy. J. Tumor Marker Oncol..

[29] Breitman T.R., E Selonick S., Collins S.J. (1980). Induction of differentiation of the human promyelocytic leukemia cell line (HL-60) by retinoic acid. Proc. Natl. Acad. Sci. USA.

[30] Rohdenburg G.L. (1918). Fluctuations in the growth of malignant tumors in man, with especial reference to spontaneous regression. J. Cancer Res..

[31] Everson T.C., Cole W.H. (1966). Spontaneous Regression of Cancer.

[32] Challis G.B., Stam H.J. (1990). The Spontaneous Regression of Cancer. A Review of Cases from 1900 to 1987. Acta Oncol..

[33] O’Regan B., Hirschberg C. (1993). Spontaneous Regression. An Annotated Bibliography.

[34] Papac R.J. (1998). Spontaneous regression of cancer: Possible mechanisms. Vivo.

[35] Livraghi T., Meloni F., Frosi A., Lazzaroni S., Bizzarri M., Frati L., Biava P.M. (2005). Treatment with Stem Cell Differentiation Stage Factors of Intermediate-Advanced Hepatocellular Carcinoma: An Open Randomized Clinical Trial. Oncol. Res. Featur. Preclin. Clin. Cancer Ther..

[36] Hendrix M.J.C., Seftor E.A., Seftor R.E.B., Kasemeier-Kulesa J., Kulesa P.M., Postovit L.-M. (2007). Reprogramming metastatic tumour cells with embryonic microenvironments. Nat. Rev. Cancer.

[37] Postovit L.-M., Margaryan N.V., Seftor E.A., Kirschmann D.A., Lipavsky A., Wheaton W.W., Abbott D.E., Seftor R.E.B., Hendrix M.J.C. (2008). Human embryonic stem cell microenvironment suppresses the tumorigenic phenotype of aggressive cancer cells. Proc. Natl. Acad. Sci. USA.

[38] Giuffrida D., Rogers I.M., Nagy A., Calogero A.E., Brown T.J., Casper R.F. (2009). Human embryonic stem cells secrete soluble factors that inhibit cancer cell growth. Cell Prolif..

[39] Costa F.F., A Seftor E., Bischof J.M., A Kirschmann D., Strizzi L., Arndt K., Bonaldo M.D.F., Soares M.B., Hendrix M.J. (2009). Epigenetically reprogramming metastatic tumor cells with an embryonic microenvironment. Epigenomics.

[40] Allegrucci C., Rushton M.D., E Dixon J., Sottile V., Shah M., Kumari R., Watson S., Alberio R., Johnson A.D. (2011). Epigenetic reprogramming of breast cancer cells with oocyte extracts. Mol. Cancer.

[41] Huang S. (2021). Reconciling Non-Genetic Plasticity with Somatic Evolution in Cancer. Trends Cancer.

[42] Versteeg R. (2014). Tumours outside the mutation box. Nature.

[43] Weinberg R.A. (2014). Coming Full Circle—From Endless Complexity to Simplicity and Back Again. Cell.

[44] Monti N., Verna R., Piombarolo A., Querqui A., Bizzarri M., Fedeli V. (2022). Paradoxical Behavior of Oncogenes Undermines the Somatic Mutation Theory. Biomolecules.

[45] Baker S.G., Kramer B.S. (2007). Paradoxes in carcinogenesis: New opportunities for research directions. BMC Cancer.

[46] Marcum J.A. (2005). Metaphysical presuppositions and scientific practices: Reductionism and organicism in cancer research. Int. Stud. Philos. Sci..

[47] Ashby J., Purchase I. (1988). Reflections on the declining ability of the Salmonella assay to detect rodent carcinogens as positive. Mutat. Res. Toxicol..

[48] Lijinsky W. (1990). Non-genotoxic environmental carcinogens. Environ. Carcinog. Rev..

[49] Greenman C., Stephens P., Smith R., Dalgliesh G.L., Hunter C., Bignell G., Davies H., Teague J., Butler A., Stevwns C. (2007). Patterns of somatic mutation in human cancer genomes. Nature.

[50] Imielinski M., Berger A.H., Hammerman P.S., Hernandez B., Pugh T.J., Hodis E., Cho J., Suh J., Capelletti M., Sivachenko A. (2012). Mapping the hallmarks of lung adenocarcinoma with massively parallel sequencing. Cell.

[51] Lawrence M.S., Stojanov P., Polak P., Kryukov G.V., Cibulskis K., Sivachenko A., Carter S.L., Stewart C., Mermel C.H., Roberts S.A. (2013). Mutational heterogene- ity in cancer and the search for new cancer associated genes. Nature.

[52] Konishi N., Hiasa Y., Matsuda H., Tao M., Tsuzuki T., Hayashi I., Kitahori Y., Shiraishi T., Yatana R., Shimazaki J. (1995). Intratumor cellular heterogeneity and alterations in ras oncogene and p53 tumor suppressor gene in human prostate carcinoma. Am. J. Pathol..

[53] Baisse B., Bouzourene H., Saraga E.P., Bosman F.T., Benhattar J. (2001). Intratumor genetic heterogeneity in advanced human colorectal adenocarcinoma. Int. J. Cancer.

[54] van Elsas A., Zerp S., van der Flier S., Krüse-Wolters M., Vacca A., Ruiter D.J., Schrier P. (1995). Analysis of N-ras Mutations in Human Cutaneous Melanoma: Tumor Heterogeneity Detected by Polymerase Chain Reaction/Single-Stranded Conformation Polymorphism Analysis. Recent Results Cancer Res..

[55] Sauter G., Moch H., Moore D., Carroll P., Kerschmann R., Chew K., Mihatsch M.J., Gudat F., Waldman F. (1993). Heterogeneity of erbB-2 gene amplification in bladder cancer. Cancer Res..

[56] Szöllösi J., Balázs M., Feuerstein B.G., Benz C.C., Waldman F.M. (1995). erBB-2 (her2/neu) gene copy number, p185her-2 overexpression and intratumor heterogeneity in human breast cancer. Cancer Res..

[57] Park S.H., Maeda T., Mohapatra G., Waldman F.M., Davis R.L., Feuerstein B.G. (1995). Heterogeneity, polyploidy, aneusomy, and 9p deletion in human glioblastoma multiforme. Cancer Genet. Cytogenet..

[58] Washington C., Dalbègue F., Abreo F., Taubenberger J.K., Lichy J.H. (2000). Loss of Heterozygosity in Fibrocystic Change of the Breast: Genetic Relationship Between Benign Proliferative Lesions and Associated Carcinomas. Am. J. Pathol..

[59] Paez J.G., Jänne P.A., Lee J.C., Tracy S., Greulich H., Gabriel S., Herman P., Kaye F.J., Lindemanm N., Boggon T.J. (2004). EGFR mutations in lung cancer: Correlation with clinical response to gefitinib therapy. Science.

[60] Zhang L., Zhou W., Velculescu V.E., Kern S.E., Hruban R.H., Hamilton S.R., Vogelstein B., Kinzler K.W. (1997). Gene expression profiles in normal and cancer cells. Science.

[61] Chanock S.J., Thomas G. (2007). The devil is in the DNA. Nat. Genet..

[62] Martincorena I., Roshan A., Gerstung M., Ellis P., Van Loo P., McLaren S., Wedge D.C., Fullam A., Alexandrov L.B., Tobio J.M. (2015). High burden and pervasive positive selection of somatic mutations in normal human skin. Science.

[63] Kan Z., Jaiswal B.S., Stinson J., Janakiraman V., Bhatt D., Stern H.M., Yue P., Haverty P.M., Bourgon R., Zheng J. (2010). Diverse somatic mutation patterns and pathway alterations in human cancers. Nature.

[64] Baker S.G. (2014). A Cancer Theory Kerfuffle Can Lead to New Lines of Research. Gynecol. Oncol..

[65] Duesberg P., Li R., Rasnick D. (2004). Aneuploidy Approaching a Perfect Score in Predicting and Preventing Cancer: Highlights from a Conference Held in Oakland, CA in January, 2004. Cell Cycle.

[66] Prehn R.T. (1994). Cancers beget mutations versus mutations beget cancers. Cancer Res..

[67] Prehn R.T. (2005). The role of mutation in the new cancer paradigm. Cancer Cell Int..

[68] Nicholson D.J. (2010). Biological atomism and cell theory. Stud. Hist. Philos. Sci. Part C Stud. Hist. Philos. Biol. Biomed. Sci..

[69] Vogelstein B., Papadopoulos N., Velculescu V.E., Zhou S., Diaz L.A., Kinzler K.W. (2013). Cancer genome landscapes. Science.

[70] Garraway L.A., Lander E.S. (2013). Lessons from the cancer genome. Cell.

[71] Bertolaso M. Philosophy of Cancer—A Dynamic and Relational View.

[72] Kamb A., Wee S., Lengauer C. (2007). Why is cancer drug discovery so difficult?. Nat. Rev. Drug Discov..

[73] Cassidy J.W., Bruna A., Uthamanthil R., Tinkey P. (2017). Chapter 4—Tumor Heterogeneity. Patient Derived Tumor Xenograft Models.

[74] Seoane J. (2017). Cancer: Division hierarchy leads to cell heterogeneity. Nature.

[75] Li R.X., Zeng R. (2009). Dynamic proteomics for investigating the response of individual cancer cells under drug action. Expert Rev. Proteom..

[76] Bhang H.E.C., Ruddy D.A., Krishnamurthy Radhakrishna V., Caushi J.X., Zhao R., Hims M.M., Singh A.P., Kao I., Rakiec D., Shaw P. (2015). Studying clonal dynamics in response to cancer therapy using high-complexity barcoding. Nat. Med..

[77] Ness R.B. (2010). Fear of Failure: Why American Science Is not Winning the War on Cancer. Ann. Epidemiol..

[78] Miklos G.L.G. (2005). The Human Cancer Genome Project—One more misstep in the war on cancer. Nat. Biotechnol..

[79] Leppert J., Patel C. (2015). Beyond the genome. Nature.

[80] Kaelin W.G. (2005). The concept of synthetic lethality in the context of anticancer therapy. Nature Rev. Cancer..

[81] Joyner M.J., Paneth N. (2019). Promises, promises, and precision medicine. J. Clin. Investig..

[82] Fojo T., Mailankody S., Lo A. (2014). Unintended consequences of expensive cancer therapeutics—The pursuit of marginal indications and a me-too mentality that stifles innovation and creativity: The John Conley Lecture. JAMA Otolaryngol. Head Neck Surg..

[83] Pierce G.B., Johnson L.D. (1971). Differentiation and cancer. In Vitro.

[84] Pierce G.B. (1983). The cancer cell and its control by the embryo. Rous-Whipple Award lecture. Am. J. Pathol..

[85] Noble D. (2007). Claude Bernard, the first systems biologist, and the future of physiology. Exp. Physiol..

[86] Soto A.M., Sonnenschein C. (2021). The cancer puzzle: Welcome to organicism. Prog. Biophys. Mol. Biol..

[87] Dennett D.C. (1995). Darwin’s Dangerous Idea.

[88] Paget S. (1989). The distribution of secondary growths in cancer of the breast. Cancer Metastasis Rev..

[89] Langley R.R., Fidler I.J. (2011). The seed and soil hypothesis revisited-The role of tumor-stroma interactions in metastasis to different organs. Int. J. Cancer.

[90] Bayir E., Sahinler M., Celtikoglu M.M., Sendemir A. (2020). Biomaterials for Organ and Tissue Regeneration.

[91] Chang H.H., Hemberg M., Barahona M., Ingber D.E., Huang S. (2008). Transcriptome-wide noise controls lineage choice in mammalian progenitor cells. Nature.

[92] Oakley E.J., Van Zant G. (2007). Unraveling the complex regulation of stem cells: Implications for aging and cancer. Leukemia.

[93] Tsuchiya M., Piras V., Choi S., Akira S., Tomita M., Giuliani A., Selvarajoo K. (2009). Emergent genome-wide control in wild-type and genetically mutated lipopolysaccharides-stimulated macrophages. PLoS ONE.

[94] Barabási A.-L., Oltvai Z.N. (2004). Network biology: Understanding the cell’s functional organization. Nat. Rev. Genet..

[95] Pearson H. (2002). Surviving a knockout blow. Nature.

[96] Bizzarri M., Cucina A., Conti F., D’anselmi F. (2008). Beyond the Oncogene Paradigm: Understanding Complexity in Cancerogenesis. Acta Biotheor..

[97] Scannell J.W., Bosley J. (2016). When quality beats quantity: Decision theory, drug discovery and the reproducibility crisis. PLoS ONE.

[98] Bizzarri M. (2017). Do new anticancer drugs really work? A serious concern, Organisms. J. Biol. Sciences..

[99] Leung C.T., Brugge J.S. (2012). Outgrowth of single oncogene-expressing cells from suppressive epithelial environments. Nature.

[100] Maffini M.V., Soto A.M., Calabro J.M., Ucci A.A., Sonnenschein C. (2004). The stroma as a crucial target in rat mammary gland carcinogenesis. J. Cell Sci..

[101] Rana B., Mischoulon D., Xie Y., Bucher N.L., Farmer S.R. (1994). Cell-extracellular matrix interactions can regulate the switch between growth and differentiation in rat hepatocytes: Reciprocal expression of C/EBP alpha and immediate-early growth response transcription factors. Mol. Cell. Biol..

[102] Huang S., Chen C.S., Ingber D.E. (1998). Control of cyclin D1, p27Kip1, and cell cycle progression in human capillary endothe- lial cells by cell shape and cytoskeletal tension. Mol. Biol. Cell.

[103] Dike L.E., Ingber D.E. (1996). Integrin-dependent induction of early growth response genes in capillary endothelial cells. J. Cell Sci..

[104] Roskelley C.D., Desprez P.Y., Bissell M.J. (1994). Extracellular matrix-dependent tissue-specific gene expression in mammary epithelial cells requires both physical and biochemical signal transduction. Proc. Natl. Acad. Sci. USA.

[105] Boudreau N., Myers C., Bissell M.J. (1995). From laminin to lamin: Regulation of tissue-specific gene expression by the ECM. Trends Cell Biol..

[106] Chen C.S., Mrksich M., Huang S., Whitesides G.M., Ingber D.E. (1997). Geometric Control of Cell Life and Death. Science.

[107] Paszek M.J., Zahir N., Johnson K.R., Lakins J.N., Rozenberg G.I., Gefen A., Reinhart-King C.A., Margulies S.S., Dembo M., Boettiger D. (2005). Tensional home- ostasis and the malignant phenotype. Cancer Cell.

[108] Colpaert C.G., Vermeulen P.B., Fox S., Harris A., Dirix L.Y., Van Marck E.A. (2003). The Presence of a Fibrotic Focus in Invasive Breast Carcinoma Correlates with the Expression of Carbonic Anhydrase IX and is a Marker of Hypoxia and Poor Prognosis. Breast Cancer Res. Treat..

[109] Martin L.J., Boyd N.F. (2008). Mammographic density. Potential mechanisms of breast cancer risk associated with mammographic density: Hypotheses based on epidemiological evidence. Breast Cancer Res..

[110] Meredith J.E., Fazeli B., Schwartz M.A. (1993). Theextracellular matrix as a cell survival factor. Mol. Biol. Cell.

[111] Boudreau N., Sympson C.J., Werb Z., Bissell M.J. (1995). Suppression of ICE and Apoptosis in Mammary Epithelial Cells by Extracellular Matrix. Science.

[112] Vosseler S., Mirancea N., Bohlen P., Mueller M.M., Fusenig N.E. (2005). Angiogenesis inhibition by vascular endothelial growth factor receptor-2 blockade reduces stromal matrix metalloproteinase expression, normalizes stromal tissue, and reverts epithelial tumor phenotype in surface heterotransplants. Cancer Res..

[113] Sternlicht M.D., Lochter A., Sympson C.J., Huey B., Rougier J.-P., Gray J.W., Pinkel D., Bissell M.J., Werb Z. (1999). The Stromal Proteinase MMP3/Stromelysin-1 Promotes Mammary Carcinogenesis. Cell.

[114] Wiseman B.S., Werb Z. (2002). Stromal Effects on Mammary Gland Development and Breast Cancer. Science.

[115] Bissell M.J., LaBarge M.A. (2005). Context, tissue plasticity, and cancer: Are tumor stem cells also regulated by the microenvironment?. Cancer Cell.

[116] Huang S. (2004). Back to the biology in systems biology: What can we learn from biomolecular networks?. Brief. Funct. Genom..

[117] Craver C.F. (2016). The explanatory power of network models. Philos. Sci..

[118] Bizzarri M., Cucina A. (2014). Tumor and the microenvironment: A chance to reframe the paradigm of carcinogenesis?. BioMed Res. Int..

[119] Noble D. (2002). Modelling the heart e from genes to cells to the whole organ. Science.

[120] Werner E. (2007). How central is the genome. Science.

[121] Longo G., Miquel P.A., Sonnenschein C., Soto A.M. (2012). Is information a proper observable for biological organization?. Prog. Biophys. Mol. Biol..

[122] Newman S.A. (2002). Developmental mechanisms: Putting genes in their place. J. Biosci..

[123] Calvert J., Parker J.N., Vermeulen N., Penders B. (2010). Systems Biology, Interdisciplinarity and Disciplinary Identity. Collaboration in the New Life Sciences.

[124] (2008). Mazzocchi, F Complexity in biology. EMBO Rep..

[125] Bizzarri M., Giuliani A., Minini M., Monti N., Cucina A. (2020). Constraints Shape Cell Function and Morphology by Canalizing the Developmental Path along the Waddington’s Landscape. BioEssays.

[126] Boogerd F.C., Bruggeman F.J., Hofmeyer J.-H.S., Westerhoff H.V. (2007). Systems Biology: Philosophical Foundations.

[127] O’Malley M.A., Dupre J. (2005). Fundamental issues in systems biology. BioEssays.

[128] Macheras P., Iliadis A. (2016). Modeling in Biopharmaceutics, Pharmacokinetics and Pharmacodynamics: Homogeneous and Heterogeneous Approaches.

[129] Melham T. (2012). Modelling, abstraction, and computation in systems biology: A view from computer science. Prog. Biophys. Mol. Biol..

[130] Green S., Şerban M., Scholl R., Jones N., Brigandt I., Bechtel W. (2017). Network analyses in systems biology: New strategies for dealing with biological complexity. Synthese.

[131] Bertolaso M., Giuliani A., De Gara L. (2010). Systems biology reveals biology of systems. Complexity.

[132] Kuhn T. (1962). Theory of Scientific Revolution.

[133] Mazzocchi F. (2015). Could Big Data be the end of theory in science?. EMBO Rep..

[134] Karpatne A., Atluri G., Faghmous J.H., Steinbach M., Banerjee A., Ganguly A., Shekhar S., Samatova N., Kumar V. (2017). Theory-guided data science: A new paradigm for scientific discovery from data. IEEE Trans. Knowl. Data Eng..

[135] Claude C.S., Longo G. (2016). The deluge of spurious correlations in big data. Found. Sci..

[136] Joyce A.R., Palsson B. (2006). The model organism as a system: Integrating ‘omics’ data sets. Nat. Rev. Mol. Cell Biol..

[137] Assmus H.E., Herwig R., Cho K.-H., Wolkenhauer O. (2006). Dynamics of biological systems: Role of systems biology in medical research. Expert Rev. Mol. Diagn..

[138] Coveney P.V., Dougherty E.R., Highfield R.R. (2016). Big data need big theory too. Philos. Trans. R. Soc. A Math. Phys. Eng. Sci..

[139] Auffray C., Nottale L. (2008). Scale relativity theory and integrative systems biology: 1: Founding principles and scale laws. Prog. Biophys. Mol. Biol..

[140] Bertolaso M., Ratti E. (2017). Conceptual Challenges in the Theoretical Foundations of Systems Biology. Syst. Biol..

[141] Boogerd F.C., Bruggeman F.J., Richardson R.C., Stephan A., Westerhoff H.V. (2005). Emergence and its place in nature: A case study of biochemical networks. Synthese.

[142] Bizzarri M., Palombo A., Cucina A. (2013). Theoretical aspects of Systems Biology. Prog. Biophys. Mol. Biol..

[143] Szent-Gyorgyi C. (1957). Bioenergetics.

[144] Adey W.R., Fröhlich H. (1988). Physiological signaling across cell membranes and cooperative influence of extremely-low frequency electromagnetic fields. Biological Coherence and Response to External Stimuli.

[145] Klink O., Hanke W., de Lima V.M.F. (2011). Gravitational Influence on an Oscillating Chemical Reaction. Microgravity Sci. Technol..

[146] Saetzler K., Sonnenschein C., Soto A. (2011). Systems biology beyond networks: Generating order from disorder through self-organization. Semin. Cancer Biol..

[147] Giuliani A., Filippi S., Bertolaso M., Wu X., Westerhoff H., De Meyts P., Kitano H. (2014). Why network approach can promote a new way of thinking in biology. Frontiers in Genetics (Systems Biology).

[148] Bailly F., Longo G. (2009). Biological organization and anti-entropy. J. Biol. Syst..

[149] Elowitz M.B., Levine A.J., Siggia E.D., Swain P.S. (2002). Stochastic gene expression in a single cell. Science.

[150] Laughlin R.B., Pines D., Schmalian J., Stojkovic B.P., Wolynes P. (2000). The middle way. Proc. Natl. Acad. Sci. USA.

[151] Noble D. (2010). Biophysics and systems biology. Philos. Trans. R. Soc. A Math. Phys. Eng. Sci..

[152] Barabasi A.L. (2007). Network medicine from obesity to the “diseasome”. N. Engl. J. Med..

[153] Soto A.M., Sonnenschein C., Miquel P.A. (2008). On physicalism and Downward Causation in Developmental and Cancer Biology. Acta Biotheor..

[154] Simon H.A. (1977). The Organization of Complex Systems. Boston Studies in the Philosophy of Science.

[155] Strange K. (2004). The end of “naive reductionism”: Rise of systems biology or renaissance of physiology?. Am. J. Physiol., Cell. Physiol..

[156] Bizzarri M., Naimark O., Nieto-Villar J., Fedeli V., Giuliani A. (2020). Complexity in Biological Organization: Deconstruction (and Subsequent Restating) of Key Concepts. Entropy.

[157] Bizzarri M., Monici M., van Loon J.J. (2015). How microgravity affects the biology of living systems. Biomed. Res. Int..

[158] Drack M., Wolkenhauser O. (2011). System approaches of Weiss and Bertalanffy and their relevance for Systems Biology today. Semin. Cancer Biol..

[159] Waddington C.H. (1935). Cancer and the theory of organizers. Nature.

[160] Needham J. (1936). New advances in the chemistry and biology of organized growth. Proc. R. Soc. Lond. B. Biol. Sci..

[161] Thompson D.A. (1917). On Growth and Form.

[162] Rossenbloich B. (2001). Outline of a concept for organismic systems biology. Semin. Cancer Biol..

[163] Goodwin B.C. (1994). How the Leopard Changed Its Spots—The Evolution of Complexity.

[164] Gilbert S.F., Sarkar S. (2000). Embracing complexity: Organicism for the 21st century. Dev. Dyn..

[165] Gilbert S.F., Barresi M.J.F. (2016). Developmental Biology.

[166] Tyler S.E.B. (2014). The Work Surfaces of Morphogenesis: The Role of the Morphogenetic Field. Biol. Theory.

[167] Løvtrup S., Løvtrup M. (1988). The morphogenesis of molluscan shells: A mathematical account using biological parameters. J. Morphol..

[168] Bissell M.J. (1981). The differentiated state of normal and malignant cells or how to define a “normal” cell in culture. Int. Rev. Cytol..

[169] Clegg J.S. (1984). Intracellular water and the cytomatrix: Some methods of study and current views. J. Cell Biol..

[170] Butcher D.T., Alliston T., Weaver V.M. (2009). A tense situation: Forcing tumour progression. Nat. Rev. Cancer.

[171] Kirson E.D., Dbalý V., Tovarys F., Vymazal J., Soustiel J.F., Itzhaki A., Mordechovich D., Steinberg-Shapira S., Gurvich Z., Schneiderman R. (2007). Alternating electric fields arrest cell proliferation in animal tumour models and human brain tumours. Proc. Natl. Acad. Sci. USA.

[172] Hammond T.G., Benes E., O’Reilly K.C., Wolf D.A., Linnehan R.M., Taher A., Kaysen J.H., Allen P.L., Goodwin T.J. (2000). Mechanical culture conditions affect gene expression: Gravity-induced changes on the space shuttle. Physiol. Genom..

[173] Bissell M.J., Barcellos-Hoff M.H. (1987). The influence of extra- cellular matrix on gene expression: Is structure the message?. J. Cell Sci..

[174] Van den Hoff A. (1988). Stromal involvement in malignant growth. Adv. Cancer Res..

[175] Waddington C.H. (1957). The Strategy of the Genes.

[176] Guo Y., Eichler G.S., Feng Y., Ingber D.E., Huang S. (2006). Towards a holistic, yet gene-centered analysis of gene expression profiles: A case study of human lung cancers. J. Biomed. Biotechnol..

[177] DiNicola S., D’Anselmi F., Pasqualato A., Proietti S., Lisi E., Cucina A., Bizzarri M. (2011). A Systems Biology Approach to Cancer: Fractals, Attractors, and Nonlinear Dynamics. OMICS A J. Integr. Biol..

[178] Vidal M., Cusick M.E., Barabási A.-L. (2011). Interactome Networks and Human Disease. Cell.

[179] Uetz P., Grigoriev A., Jorde L.B., Little P.F.R., Dunn M.J., Subramaniam S. (2005). The yeast interactome. Encyclopedia of Genetics, Genomics, Proteomics and Bioinformatics.

[180] Costanzo M., Baryshnikova A., Bellay J., Kim Y., Spear E.D., Sevier C.S., Ding H., Koh J.Y., Toufighi K., Mostafavi S. (2010). The genetic landscape of a cell. Science.

[181] Welch G.R. (2009). The ‘fuzzy’ interactome. Trends Biochem. Sci..

[182] Csermely P., Korcsmáros T., Kiss H.J., London G., Nussinov R. (2013). Structure and dynamics of molecular networks: A novel paradigm of drug discovery: A comprehensive review. Pharmacol. Ther..

[183] Ghoshal G., Barabási A.L. (2011). Ranking stability and super-stable nodes in complex networks. Nat. Commun..

[184] Bizzarri M., Giuliani A., Pensotti A., Ratti E., Bertolaso M. (2019). Co-emergence and Collapse: The Mesoscopic Approach for Conceptualizing and Investigating the Functional Integration of Organisms. Front. Physiol..

[185] Kitano H. (2002). Systems Biology: A Brief Overview. Science.

[186] Ma’ayan A. (2011). Introduction to Network Analysis in Systems Biology. Sci. Signal..

[187] Yoshida Z. (2010). Non Linear Science. The Challenge of Complex Systems.

[188] De Canete J.F., Galindo C., Garcia-Moral I. (2011). System Engineering and Automation: An Interactive Educational Approach.

[189] Huang S., Ingber D.E. (2007). A Non-Genetic Basis for Cancer Progression and Metastasis: Self-Organizing Attractors in Cell Regulatory Networks. Breast Dis..

[190] Binney J., Dowrick N.J., Fisher A.J., Newman M.E.J. (1992). The Theory of Critical Phenomena: An Introduction to the Renormalization Group.

[191] Prigogine I., Glansdorff P. (1971). Thermodynamic Theory of Structure, Stability and Fluctuations.

[192] Westerhoff H.V., Palsson B.O. (2004). The evolution of molecular biology into systems biology. Nat. Biotechnol..

[193] Schrodinger E. (1946). What is Life?. What Is Life? and Mind and Matter.

[194] Hanahan D., Weinberg R.A. (2000). The Hallmarks of Cancer. Cell.

[195] Hanahan D., Weinberg R.A. (2011). Hallmarks of cancer: The next generation. Cell.

[196] Huang S. (2013). Genetic and non-genetic instability in tumor progression: Link between the fitness landscape and the epigenetic landscape of cancer cells. Cancer Metastasis Rev..

[197] Reuveni E., Giuliani A. (2012). Emergent properties of gene evolution: Species as attractors in phenotypic space. Phys. A Stat. Mech. its Appl..

[198] Guerroui S., Deschatrette J., Wolfrom C. (2005). Prolonged perturbation of the oscillations of hepatoma Fao cell proliferation by a single small dose of methotrexate. Pathol. Biol..

[199] Zhou J.X., Aliyu M.D.S., Aurell E., Huang S. (2012). Quasi-potential landscape in complex multi-stable systems. J. R. Soc. Interface.

[200] Pisco A.O., Huang S. (2015). Non-genetic cancer cell plasticity and therapy-induced stemness in tumour relapse: ‘What does not kill me strengthens me’. Br. J. Cancer.

[201] Shen S., Clairambault J. (2020). Cell plasticity in cancer cell populations. F1000Research.

[202] Chuang H.-Y., Hofree M., and Ideker T. (2010). A decade of systems biology. Annu. Rev. Cell Dev. Biol..

[203] Marr C., Zhou J.X., Huang S. (2016). Single-cell gene expression profiling and cell state dynamics: Collecting data, correlating data points and connecting the dots. Curr. Opin. Biotechnol..

[204] Kell D.B. (2006). Systems biology, metabolic modelling and metabolomics in drug discovery and development. Drug Discov. Today.

[205] Huang S. (2011). The molecular and mathematical basis of Waddington’s epigenetic landscape: A framework for post-Darwinian biology?. Bioessays.

[206] Aldridge S., Teichmann S.A. (2020). Single cell transcriptomics comes of age. Nat. Commun..

[207] Kapuy O., He E., López-Avilés S., Uhlmann F., Tyson J.J., Novák B. (2009). System-level feedbacks control cell cycle progression. FEBS Lett..

[208] Haken H. (1977). Synergetics. Phys. Bull..

[209] Venegas J.G., Winkler T., Musch G., Vidal Melo M.F., Layfield D., Tgavalekos N., Fishman A.J., Collahan R.J., Bellani G., Scott Harris R. (2005). Self-organized patchiness in asthma as a prelude to catastrophic shifts. Nature.

[210] Tanaka G., Tsumoto K., Tsuji S., Aihara K. (2008). Bifurcation analysis on a hybrid systems model of intermittent hormonal therapy for prostate cancer. Phys. D Nonlinear Phenom..

[211] Liu X., Chang X., Liu R., Yu X., Chen L., Aihara K. (2017). Quantifying critical states of complex diseases using single-sample dynamic network biomarkers. PLoS Comput. Biol..

[212] Chen L., Liu R., Liu Z.-P., Li M., Aihara K. (2012). Detecting early-warning signals for sudden deterioration of complex diseases by dynamical network biomarkers. Sci. Rep..

[213] Goodacre R., Vaidyanathan S., Dunn W.B., Harrigan G.G., Kell D.B. (2004). Metabolomics by numbers: Acquiring and understanding global metabolite data. Trends Biotechnol..

[214] Gu Y., Lu C., Zha Q., Kong H., Lu X., Lu A., Xu G. (2012). Plasma metabonomics study of rheumatoid arthritis and its Chinese medicine subtypes by using liquid chromatography and gas chromatography coupled with mass spectrometry. Mol. Biosyst..

[215] Harrigan G.G., Goodacre R. (2003). Metabolic Profiling: Its Role in Biomarker Discovery and Gene Function Analysis.

[216] Urbanczyk-Wochniak E., Luedemann A., Kopka J., Selbig J., Roessner-Tunali U., Willmitzer L., Fernie A.R. (2003). Parallel analysis of transcript and metabolic profiles: A new approach in systems biology. EMBO Rep..

[217] Loscalzo J., Kohane I., Barabasi A. (2007). Human disease classification in the postgenomic era: A complex systems approach to human pathobiology. Mol. Syst. Biol..

[218] Müller G.B., Newman S.A. (2003). Origination of Organismal Form: Beyond the Gene in Developmental and Evolutionary Biology.

[219] Soto A.M., Sonnenschein C. (2005). Emergentism as a default: Cancer as a problem of tissue organization. J. Biosci..

[220] Kenny P.A., Bissell M.J. (2003). Tumor reversion: Correction of malignant behavior by microenvironmental cues. Int. J. Cancer.

[221] Kolodkin A., Boogerd F.C., Plant N., Bruggeman F.J., Goncharuk V., Lunshof J., Moreno-Sanchez R., Yilmaz N., Bakker B.M., Snoep J.L. (2012). Emergence of the silicon human and network targeting drugs. Eur. J. Pharm. Sci..

[222] Amson R., Karp J.E., Telerman A. (2013). Lessons from tumor reversion for cancer treatment. Curr. Opin. Oncol..

[223] Pisanu M.E., Noto A., De Vitis C., Masiello M.G., Coluccia P., Proietti S., Giovagnoli M.R., Ricci A., Giarnieri E., Cucina A. (2014). Lung Cancer Stem Cell Lose Their Stemness Default State after Exposure to Microgravity. BioMed Res. Int..

[224] Tripathi A., Kashyap A., Tripathi G., Yadav J., Bibban R., Aggarwal N., Thakur K., Chhokar A., Jadli M., Sah A.K. (2021). Tumor reversion: A dream or a reality. Biomark. Res..

